# Delayed Airway Obstruction after Internal Jugular Venous Catheterization in a Patient with Anticoagulant Therapy

**DOI:** 10.1155/2011/359867

**Published:** 2011-12-21

**Authors:** Pei-Ju Wu, Siu-Wah Chau, I-Cheng Lu, Hung-Te Hsu, Kuang-I Cheng

**Affiliations:** ^1^Department of Anesthesiology, Kaohsiung Medical University Hospital, 100, TzYou 1st Road, Kaohsiung 807, Taiwan; ^2^Department of Anesthesiology, Kaohsiung Municipal Hsiao-Kang Hospital, Kaohsiung 812, Taiwan; ^3^Department of Anesthesiology, Faculty of Medicine, College of Medicine, Kaohsiung Medical University, Kaohsiung 807, Taiwan

## Abstract

Delayed onset of neck hematoma following central venous catheterization without arterial puncture is uncommon. Herein, we present a patient who developed a delayed neck hematoma after repeated attempts at right internal jugular venous puncture and subsequent enoxaparin administration. Progressive airway obstruction occurred on the third day after surgery. Ultrasound examination revealed diffuse hematoma of the right neck, and fibreoptic examination of the airway revealed pharyngeal edema. After emergent surgical removal of the hematoma, the patient was extubated uneventfully.

## 1. Introduction

Long-term oral anticoagulant is indicated in patients with chronic atrial fibrillation at high risk of life-threatening cardiovascular disease. For the patient undergoing a major surgery intervention, a bridging therapy of low-molecular-weight heparin (LMWH) in place of oral anticoagulants is suggested to avoid the disaster of perioperative thromboembolism and pulmonary emboli [1–3]. However, the potential consequences of intraoperative or postoperative wound bleeding may complicate the success of the admission. Here, we present a patient undergoing spinal surgery with oral warfarin and digoxin for chronic atrial fibrillation and impaired left ventricle function. Bridging therapy was administered perioperatively to prevent venous thromboembolism. However, a delayed airway obstruction caused by unexpectedly severe neck hematoma manifested on the 3rd postoperative days, and it was closely associated with failure of the right internal jugular vein catheterization.

## 2. Case Presentation

**Figure 1:**
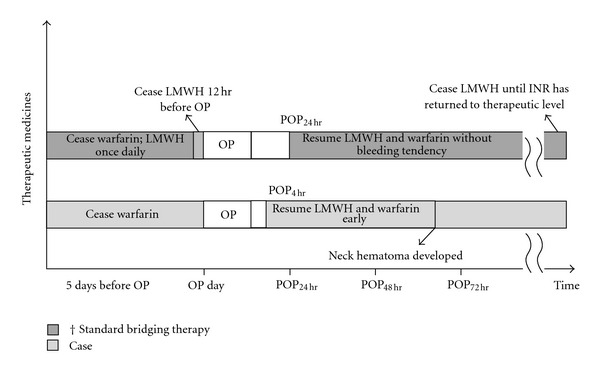
Protocol of standard bridging therapy and medications in patients with low thromboembolism risk/high bleeding risk. LMWH is continued until the INR has returned to the therapeutic level. LMWH: low molecular weight heparin; OP: operation; POP: postoperatively; INR: international normalized ratio.

A 61-year-old male (weight, 83 kilograms) suffering from recurrent lumbar herniation of intervertebral disc (HIVD) was scheduled for laminectomy under general anesthesia. He had been treated for chronic atrial fibrillation with oral warfarin and digoxin for several years and bridging therapy of Enoxaparin instead of warfarin perioperatively for its low thromboembolism risk/high bleeding risk [4–6]. The postoperative bridging therapy regimen proposed by the cardiologist was 60 mg enoxaparin, a LMWH, administered subcutaneously twice daily and was continued until the INR returned to therapeutic levels (Figure 1). On the day of surgery, coagulopathy test results, including international normalized ratio (INR = 1.13), prothrombin time (PT; PT_p_/PT_c_ = 12.2/11.6), activated partial thromboplastin time (aPTT; PTT_p_/PTT_c_ = 29.5/28.7), and platelet count (169,000 cells/mL) were within normal limits.

Fentanyl 2 *μ*g·kg^−1^, Thiamylal 5 mg·kg^−1^, and Atracurium 0.5 mg·kg^−1^ were administered for induction of general anesthesia, and 2.5% sevoflurane in oxygen was used for maintenance. After endotracheal intubation, the patient's head was turned to the left 45 degrees with a neutral neck position and internal jugular venous catheterization was attempted by approaching the apex of the triangle formed by the two heads of the sternocleidomastoid muscle and the clavicle as a landmark. An 18-gauge (G) introducer needle was successfully inserted into the jugular vein alongside the 22 G finder needle but failed to indwell the guide-wire at the first attempt. Another three attempts to puncture the right internal jugular vein and one attempt on the right subclavian vein all failed. There was no aspiration of blood from carotid or subclavian arteries. Digital compression of needle puncture sites was applied for five minutes to cease potential bleeding. In addition, there was no bruising or ecchymosis in the right neck. Subsequently, a double-lumen central venous catheter was successfully inserted into the left internal jugular vein at the first attempt under ultrasound guidance.

**Figure 2:**
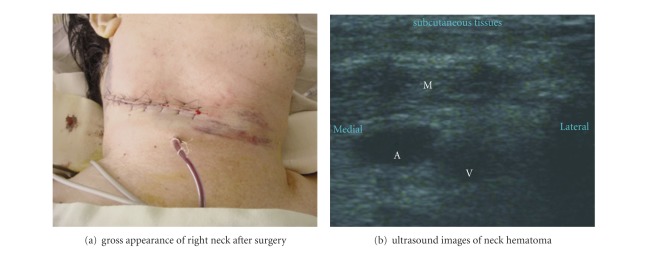
Delayed right neck hematoma. (a) Gross appearance of the neck after surgery. (b) Ultrasound image of right neck showed edematous muscles and swelling connective tissues. No thrombus in the jugular vein: common carotid artery (A), internal jugular vein (V), and edematous muscle layers (M).

The patient was transferred to the intensive care unit (ICU) for postoperative care and after an uneventful interval, to the ordinary ward. As shown in Figure 1, bridging therapy with enoxaparin 0.75 mg/kg was subcutaneously administered 4 hrs postoperatively and then twice daily for the next 2 days. Oral warfarin was administered starting again on the next morning. The hemostasis data of INR and aPTT_p/c_ showed 0.99 and 31.9/29.2 on the next postoperative day, respectively. However, it was noticed that the patient developed progressive dyspnea and stridor for his right neck with massive swelling about 52 hours after enoxaparin administration; after then, the hemostasis data revealed INR 1.23, PTT_p/c_ 42.9/28.8, hemoglobin 9.1 g/dL, and platelet 134,000 cells/mL. Ultrasound examination of the swollen area of the right neck showed diffuse hematoma (Figure 2(a)). The oropharynx and hypopharynx were edematous as examined by an otolaryngologist, and emergent surgical decompression was recommended. Due to difficult masking and airway obstruction by edematous pharyngeal tissues, awake nasotracheal intubation was performed with a fiberoptic bronchoscope (Olympus ENF XP 4.5 mm; Olympus, Tokyo, Japan) loaded with an endotracheal tube (RAE, ID 7.0 mm). General anesthesia was maintained with sevoflurane in oxygen. Surgical exploration evacuated hematomas and decompressed soft tissue and muscle groups in the right neck. After operation, a drainage tube was placed over the right neck (Figure 2(b)). The patient was transferred to the neurologic intensive care unit for mechanical ventilation. Four days later, the patient was extubated and transferred to the ordinary ward without further complications and finally discharged from the hospital uneventfully.

## 3. Discussion

Airway obstruction secondary to neck hematoma is an emergent and potentially fatal condition. It may occur after surgical procedures on the neck such as carotid, thyroid, or cervical spine surgery [7–10]. Otherwise, procedures of central venous catheterization easily damage large vessels that cause airway compromise for hematoma on prevertebral [11], mediastinal [12], and retropharyngeal spaces [13]. Most of the airway obstructive episode is associated with hematoma immediately or within several hours after venous catheterization; however, airway compromise by delayed onset of bulky hematoma is rare. The delayed onset hematoma is easily overlooked initially, and it may cause severe upper airway obstruction to a life-threatening situation requiring immediate airway management. Urgent evacuation was needed to decompress the hematoma in this patient.

As the patient was classified to have low thromboembolism risk (atrial fibrillation with congestive heart failure) with high bleeding risk (lumbar spinal surgery) [4], prevention of postoperative wound bleeding over thromboembolism was the therapeutic principal. Hence, administration of anticoagulants to the patient was allowed to deviate from guidelines of standard bridging therapy [4, 6, 14] from withdrawal of warfarin for 5 days without LMWH preoperatively and from early subcutaneous LMWH dose of 0.75 mg/kg postoperatively. However, unexpectedly delayed onset neck hematoma occurred. In this presentation, when a PTT is prolonged in isolation, heparin therapy is more probable than warfarin, because warfarin will first prolong the PT and later the aPTT as well. Therapeutic enoxaparin dose once daily perioperatively with the first postoperative dose administered 12–24 hrs after surgery greatly increases the occurrence of major bleeding [15]. For low-dose LMWH either within 6 h or 12 to 24 h after major orthopedic surgery, the risk for bleeding is higher in patients who received LMWH closer to surgery [16]. It demonstrated that subcutaneous enoxaparin in either early administration or delayed withdrawal from this patient resulted in the episode of bleeding.

Our case presentation revealed failed attempts of venous puncture and failed wire advancement initially. As reported by Eisen et al. [17], the incidence of catheterization complications increases from one puncture (17%) to attempts of 2 punctures (28%) and increases up to 54% after three or more attempts. Therefore, central venous catheterization under ultrasound guidance has gained increasing popularity for easy viewing of vessels and real-time guidance of catheter insertion. Ultrasound improves the success rate of internal jugular or subclavian vein catheterization, reduces the access time, decreases puncture attempts, and reduces or even abolishes unintentional arterial dilatation. Although wire advancement through the jugular vein was not guided by ultrasound, the vessel wall of the jugular vein might have been injured during a failed dilator insertion in this patient. Therefore, to avoid unintentional mishaps, central venous catheterization under ultrasound guidance is highly suggested.

In conclusion, delayed airway obstruction is a rare but potentially lethal complication in internal jugular venous catheterization, particularly in patients undergoing anticoagulant therapy. If a patient undergoing anticoagulant therapy requires catheterization of the internal jugular vein, an anatomical landmark incorporating ultrasound guidance is highly recommended.
